# Oscillation of an Anuran Hybrid Zone: Morphological Evidence Spanning 50 Years

**DOI:** 10.1371/journal.pone.0052819

**Published:** 2012-12-26

**Authors:** Jean-Sébastien Roy, David O’Connor, David M. Green

**Affiliations:** Redpath Museum, McGill University, Montréal, Québec, Canada; Uppsala University, Sweden

## Abstract

**Background:**

The hybrid zone between the primarily forest-dwelling American toad, *Anaxyrus americanus*, and the prairie-adapted Canadian toad, *A. hemiophrys*, in southeastern Manitoba is known to have shifted its position during the past 50 years. Hybrid zones are areas of interbreeding between species and their movement across a landscape should reflect their underlying dynamics and environmental change. However, empirical demonstrations of hybrid zone movements over long periods of time are rare. This hybrid zone is dominated by individuals of intermediate morphology and genetic composition. We sought to determine if it had continued to move and if that movement was associated with shifts in habitat, as predicted.

**Methodology/Principle Findings:**

We used variation in the toads’ most diagnostic morphological feature, the separation between their interorbital cranial crests, to determine the geographic position of the hybrid zone center at four times between 1960 and 2009 using maximum likelihood methods. The hybrid zone center moved west by 38 km over 19 years and then east again by 10 km over the succeeding 29 years. The position of the hybrid zone did not track either the direction or the magnitude of movement of the forest-prairie habitat transition over the same time period.

**Conclusions/Significance:**

This is the first reported evidence of oscillation in the position of a hybrid zone. The back and forth movement indicates that neither species maintains a selective advantage over the other in the long term. However, the movement of the hybrid zone was not bounded by the breadth of the habitat transition. Its oscillation suggests that the hybrid zone is better described as being elastically tethered to the habitat transition.

## Introduction

Hybrid zones, which are regions where species meet and interbreed, may move across a landscape [1] but this is not so easily documented empirically. Few studies of hybrid zones repeatedly sample with sufficient intensity over a sufficiently long a period of time to be able to clearly identify movement [2], [3]. Buggs [1] only identified 23 studies that directly demonstrated moving hybrid zones plus 16 others that showed genetic characteristics consistent with past hybrid zone movement. To this list may also be added studies of hybrid zones among warblers and baboons [4], [5], [6] as well as the hybrid zone between the American toad, *Anaxyrus* ( = *Bufo*) *americanus*, and the Canadian toad, *A. hemiophrys*, in southeast Manitoba, which evidently shifted westward between 1968 and 1979 [7].

Hybrid zones between species should move where differences in the strength of selection against the hybrids and the extent of dispersal of the hybridizing species allow one parental form to outcompete the other [8], [9], [10]. Where the hybridized individuals within a hybrid zone exhibit lesser fitness than pure individuals of the parental taxa to either side, the resulting “tension zone” is a narrow cline whose position is strongly affected by the balance between selection against hybrids and the differential immigration of parental forms [10]. Alternatively, where hybrids have greater fitness than the parental forms within the habitat transition that lies between them [11], the resulting “ecotonal” hybrid zone is maintained by selection against immigrants [12] and should shift location only in relation to shifts in the position and width of the habitat transition of the ecotone [13], [14]. Movements of hybrid zones relative to surrounding habitat are therefore expected to be directly related to the zones’ underlying dynamics [15], [16].

The hybrid zone between the toads, *A. americanus* and *A. hemiophrys*, has the characteristics of a habitat-bounded “ecotonal” hybrid zone. The species differ substantially in mating call, habitat preference, morphology and allozymes, and their hybrid zone is predominantly populated by morphologically and genetically intermediate individuals [7], [17], [18], [19]. There is no evidence of genetic disequilibrium or heterozygote deficiency within the hybrid zone populations, consistent with an absence of strong selection against hybrids and low immigration of parental individuals into the zone [7]. Although the relative fitness of the parental types and hybrids in their respective habitats has not been directly investigated, Cook [18] approached this issue by raising experimental crosses of all possible parental combinations in natural ponds located east (96.28°W longitude) and west (96.57°W longitude) of the hybrid zone center along Hwy 1 in Manitoba in 1968. Cook’s data indicated that the offspring of *A. hemiophrys* mothers survived through to metamorphosis better in the western pond than in the eastern pond, but no other significant differences in tadpole survival were detectable between sites for any other cross. All of this is consistent with expectations for a dispersal-independent, ecotonal hybrid zone in which freely back-crossing populations are relatively unaffected by immigration from either side. The genetic composition of toad populations across the hybrid zone is strongly correlated with their morphological variation [7], indicating that morphology can be used as effectively as genetic information to assess the shape and position of the hybrid zone.

The *A. americanus* × *hemiophrys* hybrid zone is known to have moved westward before, into the geographical range of *A. hemiophrys* [7]. If it has continued to move in the same direction since it was last examined then the center of the cline between animals with morphologies like *A. americanus* and animals with morphologies like *A. hemiophrys* should be located further west. Furthermore, the hybrid zone spans the habitat transition between the eastern forest favored by *A. americanus* and the western prairie favored by *A. hemiophrys*, [7], [19]. As such, any movement of its geographic position should be bounded by the breadth and location of the forest-prairie habitat transition. Therefore, mapping changes in position of the hybrid zone in relation to the position and breadth of the habitat transition should demonstrate a high level of coincidence between the two.

## Materials and Methods

### Ethics Statement

All procedures with animals were authorized under Manitoba Scientific Wildlife Permits WB07790 and WB06009, and McGill University Animal Use Protocol 4569.

### Specimens

**Figure 1:**
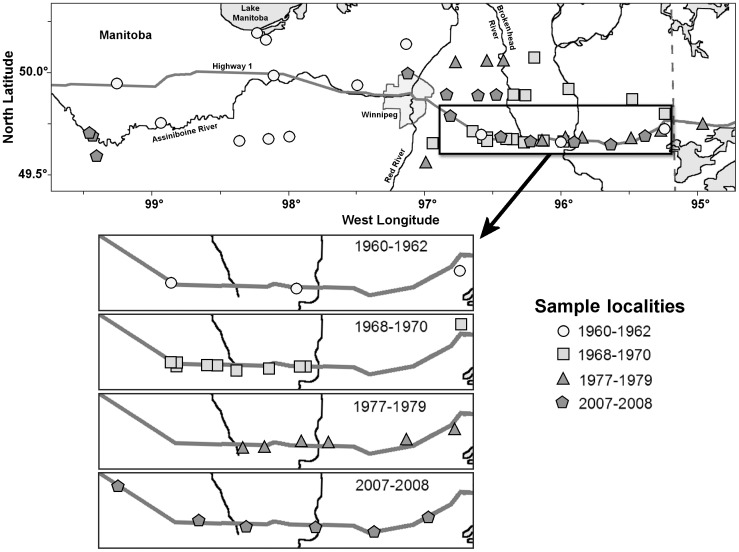
Sample sites in southeastern Manitoba and northwestern Ontario for collections of Canadian Toads, *Anaxyrus hemiophrys*, and American Toads, *A*. *americanus*, and their hybrids.

We studied toads along a roughly 400 km long, east-west transect across southern Manitoba from Whiteshell (95.91°W Longitude), near the Ontario border in the east, to Oak Lake (100.64° W Longitude), west of the city of Brandon in the west (Fig. 1). As in previous studies [7], [18], [19], the transect largely followed the route of Hwy 1 (49.65° –49.97°N Latitude) through the hybrid zone. Toads were captured from across the transect during spring in 2007 and 2008. Toad choruses were located acoustically and animals were caught either by hand or with a dipnet. We collected a minimum of five and a maximum of 30 specimens per site. All but 18 specimens, selected at random and retained as museum vouchers, were marked to avoid duplicate data if recaptured and released. The toads kept as vouchers (Appendix S1) were humanely sacrificed through oral application of benzocaine ointment, fixed in 10% formalin and stored in 70% ethanol.

In addition to our new specimens from 2007 and 2008, we used 1,276 preserved specimens taken from across the transect and dating back to 1962 from the collections of the Canadian Museum of Nature (Appendix S1) that had been previously studied [7], [18], [19]. For analysis, we distinguished between the four time periods during which collections had been made: 1960–1962 (*n* = 430), 1968–1970 (*n* = 796), 1978–1979 (*n* = 130) and 2007–2008 (*n* = 183). Each of these encompassed 10 or more sites, with at least 5 specimens per site, over a period of 4 years or less. We also examined reference specimens of *A. americanus* (*n* = 107) from Québec and Ontario and *A. hemiophrys* (*n* = 78) from Alberta and Saskatchewan (Appendix S1).

### Measurements

We photographed all toads, both living and preserved, in dorsal aspect at a resolution of 4 megapixels per image using a digital camera mounted in a standardized apparatus to ensure consistency. We included a ruler in each picture for scale. Morphological characters were measured from the standardized photographs using *Image Tool* 3.0 [20]. The use of photographs allowed us to measure animals without necessarily killing them, keep a standardized record of all specimens and measure small characters with increased precision from enlarged images.

**Figure 2:**
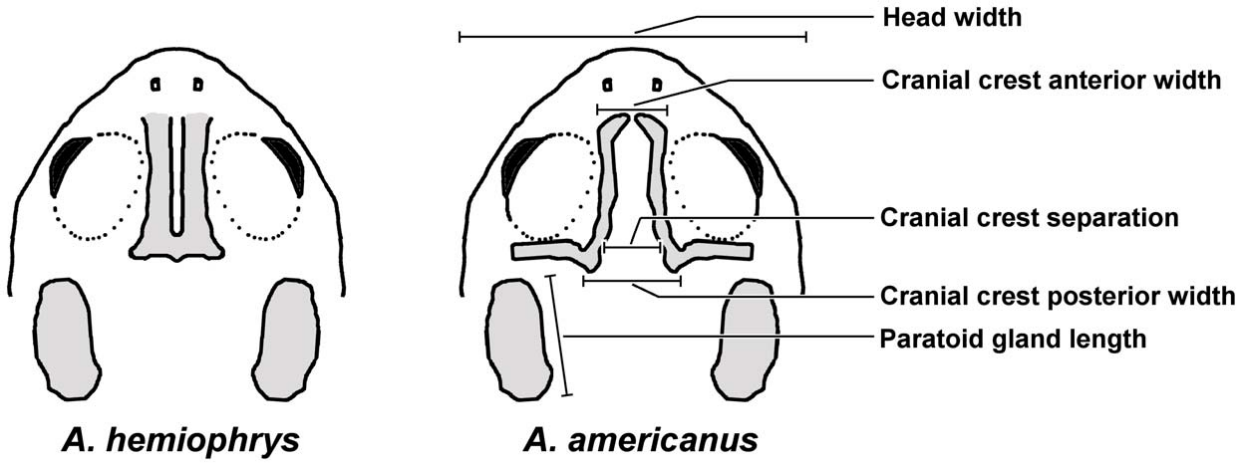
Cranial crests of Canadian Toad, *Anaxyrus hemiophrys*, and American Toad, *A*. *americanus*, illustrating the morphological measurements used in this study.

The principle morphological features distinguishing *A. americanus* and *A. hemiophrys* are their bony cranial crests (Fig. 2), which can be readily studied in preserved specimens [18]. In *A. americanus*, two distinct L-, or J-, shaped crests start at the nostril region and run more or less in parallel between the eyes, then turn at right angles to run behind the back of the eyes. The crests in *A. hemiophrys*, however, are fused between the eyes, forming a boss that may or may not be grooved in the middle. The ridges behind the eyes are less apparent, often discontinuous and may not be fused to the interorbital crests.

Following Cook [18], we used six discriminating characters (Fig. 2): Cranial Crest Anterior (CCA) – the distance across the anterior part of the cranial crests, Cranial Crest Posterior (CCP) – the distance across the posterior part of the cranial crests, Cranial Crest Separation (CCS) – the interior distance between the crests, Head Width (HW) – the width of the head at the tympani, Parotoid Gland Length – the length of parotoid glands, both left (PGL) and right (PGR), and Snout-vent Length (SVL). For consistency, only adult males of SVL >43 mm were used for analysis. We normalized data to control for variation in individual size by scaling each measurement relative to an idealized SVL of 50 mm. We performed a discriminant analysis using JMP statistical software with the data from the reference samples to determine the strength of each of the morphological variables to distinguish between the two species of toads according to Wilks’ Lambda statistic [21]. Unlike the previous studies of this hybrid zone [7], [18], [19], we analyzed the clinal structure of each variable separately and did not use the discriminant analysis to derive an overall discriminant function incorporating coefficients for all variables.

Because alcohol preserved specimens are known to shrink over time [22], we compensated for such preservation artifacts in two ways. First, we computed a coefficient of shrinkage for preserved specimens that enabled use of data from both live and preserved animals. To do this, we re-photographed 13 specimens retained as vouchers after they had been preserved in ethanol after four months and again after four years. Since body length has been shown to stabilize after less than two months of preservation [23], [24], [25], we considered four months to be adequate time in preservative to observe an effect and four years enough time to test if the shrinkage had stabilized. In line with these previous studies, we expected some characters, such as SVL, to shrink significantly in preservative and others, such as features of the bony cranial crests, to show little, if any, change [22]. Second, we did not analyze morphological characters through time directly. Instead, we analyzed the pattern of relative morphological change geographically across the hybrid zone independently for each time period, which ensured that all of the specimens for any one of these analyses were in a similar state of preservation. We then used the results of these analyses to investigate the position and width of the cline over time. With these procedures, we were confident that preservation artifact would not be allowed to affect our results.

### Mapping

We converted all geo-coordinates, in decimal degrees longitude and latitude, onto a gnomonic projection [26] in order to reduce distortion and obtain x and y map coordinates in meters that could be mapped without the problems induced by spanning UTM zones. We located the center of projection at Winnipeg city center (97.14°W, 49.88°N). As the midpoint of the transition zone between the two species runs north-south [19], we mapped the geographic position of the cline center in terms of the *x* positional variable, i.e. longitude.

### Habitat Characterization

**Figure 3:**
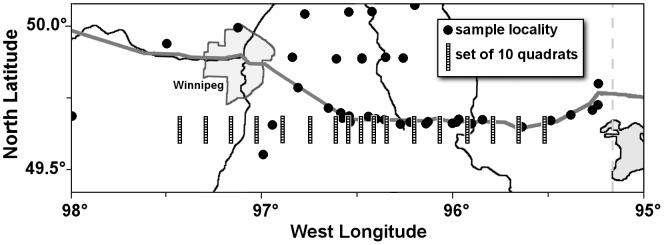
Locations of the 17 sets of 1 km^2^ quadrats across southeastern Manitoba used to determine extent of forest cover from 1∶15,000 scale topographic maps. Sample localities for toads in the vicinity of the quadrats are depicted as black dots.

To map the geographic position the forest-prairie habitat transition in the region of the toads’ hybrid zone, we estimated the relative extent of forest cover based on 1∶50,000 scale topographic maps. We used map sheets dated 1948–1957, 1969–1974 and 1980–1990, and digital maps dated 1998–2001, obtained from the Centre for Topographic Information, Natural Resources Canada. We estimated area of forest cover by placing 17 sets of 10 1-km^2^ quadrats on the maps between 49.61°N and 49.70°N latitude, with the northernmost quadrat in each set placed on or slightly north of Hwy 1(Fig. 3). The quadrats in each set were lined up north-south and the sets were placed every 10 km between longitudes 95.50°W and 97.43°W to span the forest-prairie habitat transition, with two additional sets intercalated on either side of the centermost set so that the central region could be sampled on a finer scale. With the paper map sheets, we determined the extent of forest cover in each quadrat by dividing it into 100 squares of one hectare each and counting the number of hectares filled by forest, by other terrain or by bodies of water. With the digital maps, we measured the areas depicted as forest, other terrain and bodies of water in each quadrat directly using *ArcGis 9.3*. To calculate the relative extent of forested vs. unforested land in each quadrat, we subtracted areas occupied by water bodies and calculated the total area of forest relative to the remaining land to derive a value from 0 to 1, where 0 indicated a completely unforested quadrat and 1 indicated a completely forest-covered quadrat. We averaged these values over the ten quadrats within each set to derive landscape habitat values for each of 17 locations spanning the forest-prairie transition for each of the four time periods represented by the maps.

### Cline Characterization and Analysis

In all, we analyzed eight east-west clines using the program *Analyze 1.3* [27] after the manner of Macholán et al. [28]: four clines in toad cranial crest morphology spanning the hybrid zone during each of the time periods 1960–1962, 1968–1970, 1978–1979 and 2007–2008, and four clines in forest cover spanning the forest-prairie habitat transition during each of the time periods 1948–1957, 1969–1974, 1980–1990 and 1998–2001. To determine the position of the center of each cline, we plotted the relative morphology or habitat variable on the *y* axis against the positional variable, longitude, on the *x* axis and used a cubic spline procedure to smooth the curve. We could then determine center of the cline as the geographic location of the mid-value of the steepest region of the curve. To compare cline shapes, we fit them into the sigmoid model of a cline [27] which we defined by the hyperbolic tangent function:
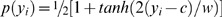
where *p*(*y_i_*) is the value, from 0–1, of character *y* at *i*th site, *c* is the position of the cline center, (*y_i_ – c*) is the distance of the *i*th site from *c*, and *w* is the cline width, derived as the inverse of the maximum slope of the curve [28], [29]. We used this particular model as it is defined by only the two parameters, *c* and *w*, in addition to the trait value at each site, *p*(*y_i_*) [28]. Our possible use of symmetrical or asymmetrical stepped models as alternatives [28] would have required inclusion of additional parameters describing selection or dispersal for which we did not have information.

We determined concordance between clines by creating log-likelihood (LogL) profiles for forest cover and morphology [28], [29]. For each cline, we kept the center position, *c*, fixed and explored the likelihood surface for all possible values of center position, keeping other parameters, *w* and *p*(*y_i_*), free to vary. To find the maximum likelihood values for all putative positions of the cline center, we applied a Markov chain Monte Carlo (MCMC) method, with 500 to 1000 iterations depending on the number of data points, based on the modified Metropolis–Hastings algorithm [30], [31] as implemented in *Analyse 1.3*. As suggested by Szymura and Barton [32], we used 2-unit log-likelihood support limits, *i.e.* Lmax –2(LogL), to approximate 95% confidence intervals [28].

We determined levels of coincidence between clines by comparing LogL profiles [28]. First, we built a composite likelihood profile by summing likelihood profiles of the two clines to be compared. Then we summed the maximum LogL values for each of the two original profiles ( = LLΣ) and compared that to the maxLogL of the composite profile ( = ΣLL). If clines are not concordant, the two values should be significantly different, which we determined with a Likelihood Ratio Test (LRT) conducted on the LLΣ and ΣLL values, with *n*-1 degrees of freedom, where *n = *2 clines [28]. The null hypothesis was that there was no difference between clines. We calculated the likelihood ratio, *R*, according to the formula, *R* = 2 (LLΣ – ΣLL), where *R* follows a χ^2^ distribution with 1 degree of freedom. We could then calculate *P* values for the significance of the difference. Summing LogL for different traits assumes that they are independent [29], which is the case here because the different LogL profiles represent different sampling periods. We tested the coincidence of clines in morphological characters of the toads against the cline in forest cover nearest in time to when the toads had been caught. We applied the sequential Bonferroni method for correcting levels of significance for multiple independent tests [33] to the results of each of the three types of comparisons between clines: morphology vs. morphology, forest cover vs. forest cover, morphology vs. forest cover.

## Results

### Morphology

The discriminant analysis showed CCS (Wilks’ Lambda = 0.105) to be by far the most significant of the seven morphological variables for differentiating the reference samples of the two species (Table 1). CCS averaged 3.1±0.6 (S.D.) mm (range = 1.8 to 4.4 mm) in the *A. americanus* reference specimens and 0.0±0.1 mm (range = 0.0 to 0.7 mm) in the *A. hemiophrys* reference specimens. It was the only variable to discriminant between the species with no misclassifications. The next most significant variable was CCP (Wilks’ Lambda = 0.533), which averaged 6.2±0.9 (S.D.) mm (range = 4.3 to 8.4 mm) in the *A*. *americanus* reference specimens and 4.7±0.9 mm (S.D.) (range = 3.1 to 6.9 mm) in the *A. hemiophrys* reference specimens, with near 20% misclassifications. For all other variables, Wilks’ Lambda was greater than 0.9. Therefore only CCS was retained for further analysis.

**Table 1:** Discriminant analysis of morphological characters.

	*F*	Wilks’ Lamdba	*R*	% misclassified
Cranial Crest Anterior (CCA)	3.92	0.976	0.155	41.4
Cranial Crest Posterior (CCP)	140.39	0.533	0.684	19.8
Cranial Crest Separation (CCS)	1366.65	0.105	0.946	0.0
Head Width (HW)	0.74	0.995	0.068	40.2
Parotoid Gland Left (PGL)	0.05	0.959	0.202	43.7
Parotoid Gland Right (PGR)	<0.01	1.000	0.005	50.3
Snout-vent Length (SVL)	1.22	0.992	0.087	45.6

Discriminant analysis of morphological characters tested for their ability to distinguish between reference samples of American Toads, *Anaxyrus americanus*, and Canadian Toads, *A. hemiophrys*.

Shrinkage artifact of ethanol preservation occurred to a varying extent among the different morphological variables. However the bony characters of the cranial crests, such as CCS, showed no evidence of being affected by preservation. CCS did not change significantly either after 4 months preservation (Wilcoxon signed rank test [two-tailed], *n = *13; W = 3.5, *P* = 0.734) or after 4 years preservation (W = 11.5, *P* = 0.203). We therefore used CCS in our analyses without adjustment.

### Hybrid Zone

**Figure 4:**
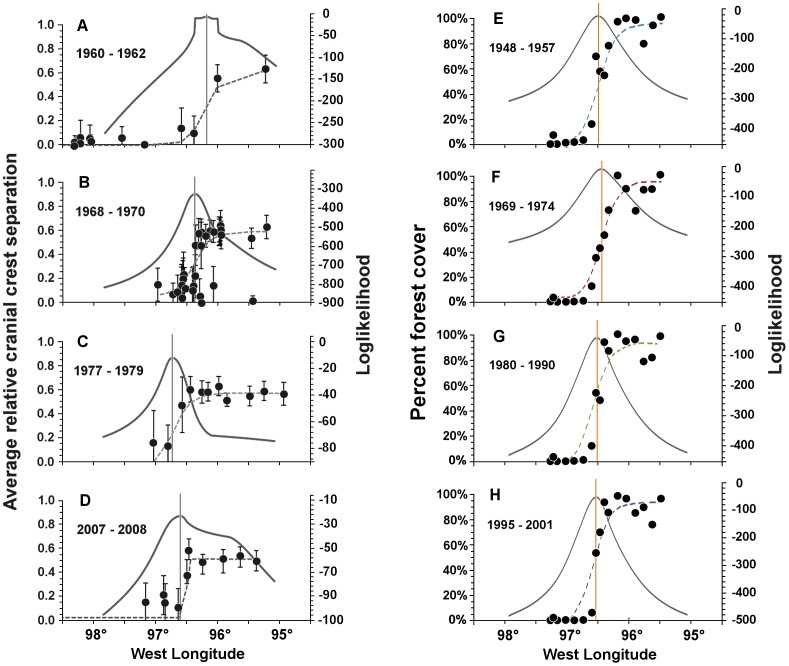
Position and shape of clines in southeastern Manitoba in (A–D) cranial crest separation in samples of Canadian Toad, *Anaxyrus hemiophrys*, American Toad, *A*. *americanus*, and their hybrids at different times between1960 and 2008, and (E–F) the forest-prairie habitat transition at different times between 1948 and 2001. Dashed lines are the best fit shapes of clines. Solid lines depict likelihood profiles. The vertical line represents the position of the cline center.

Values of CCS among toads from about 95.5°W to 96.0°W longitude averaged 2.94±0.03 (S.D.) mm in 1960–1962 and 3.04±0.05 mm in 2007–2008, well within the normal ranges of values seen in the *A. americanus* reference samples. To the west, between 98.0°W and 99.0°W longitude, CCS values were less than 0.50 mm in all years, as is typical of the *A. hemiophrys* reference sample. In the central region of the transect, CCS values shifted from those typical of *A. americanus* those typical of *A. hemiophrys* in a cline that did not significantly change in shape over the years examined (Fig. 4). The cline in 1960–1962 had a width of 29 km (LogL support limits = 20–38.5 km), though relatively sparse sampling in 1960–62 may have influenced our estimation of the zone’s width during that time period since we found it to be less than 5 km wide during the other time periods. We estimated it to be 0.5 km (0–1 km) wide in 1968–70, 0.5 to 4 km (0.5–21 km) wide in 1977–1979 and 0.5 to 1 km (0.5–8.5 km) wide in 2007–2008. Few individuals with pure parental species characteristics were present within the center of the hybrid zone.

The center of the cline in CCS values shifted significantly over the past 50 years (Fig. 4). In 1960–1962, it was located 68±4 km east of Winnipeg city center (96.19±0.06°W). It shifted some 13 km west to lay 55±2 km east of Winnipeg city center (96.37±0.02°W) by 1968–1970. By 1977–1979, it lay a further 25 km west at 30±4 km east of Winnipeg city center (96.72±0.06°W) but between then and 2007–2008, if shifted east again by about 10 km to lay 40±6 km east of Winnipeg city center (96.58±0.08°W). These positions were all significantly different from each other in pair-wise comparisons with sequential Bonferroni correction (Table 2).

**Table 2:** Pair-wise tests of coincidence among morphological clines of cranial crest separation in toads.

		Toad cranial crest separation				Forest cover			
		1960–62	1968–70	1977–79	2007–08	1948–57	1969–74	1980–90	1995–2001
**Cranial crest separation**	**1960–1962**	–	6.74	82.53	25.02	48.04	–	–	–
	**1968–1970**	0.009*^†^	–	73.88	18.28	–	11.19	–	–
	**1977–1979**	<0.001*^†^	<0.001*^†^	–	4.22	–	–	26.47	–
	**2007–2008**	<0.001*^†^	<0.001*^†^	0.040*^†^	–	–	–	–	2.06
					–				
**Forest cover**	**1948–1957**	<0.001*^†^	**-**	**-**	–	–	4.44	0.11	1.53
	**1969–1974**	–	0.001*^†^	–	–	0.035*	–	8.08	12.70
	**1980–1990**	–	–	<0.001*^†^	–	0.742	0.004*^†^	–	0.32
	**1995–2001**	–	–	–	0.151	0.216	<0.001*^†^	0.57	–

Pair-wise tests of coincidence among morphological clines of cranial crest separation in toads during different time periods, among habitat clines between forest and prairie during different time periods, and between clines in toad morphology and habitat at most closely comparable time periods. Likelihood Ratio Test (LRT) values are above the diagonal and probability (*P*) values are below the diagonal. Asterisks (*) indicate significant difference at α = 0.05. Daggers (^†^) indicate significant difference following sequential Bonferroni correction for multiple independent tests.

### Habitat Transition

The likelihood profiles for forest cover all featured only one sharp peak, indicating that the forest-prairie center position was restrained to a small region (Fig. 4). The forest cover transition zone appeared to decrease in width over time, from 31±9.5 km wide on the 1948–57 map to 25.5±9 km wide on the 1969–74 map, 17±5 km wide on the 1980–90 map and just 10.5±4 km wide on the most recent, 1995–2001 map (Fig. 4). It also shifted its position during the same time period. During 1948–1957, the center of the transition was 46±3 km east of Winnipeg (96.50±0.04°W) but by 1969–1974, it had shifted east by about 4 km to lie at 50±3 km east of Winnipeg (96.44±0.03°W). The transition center subsequently moved west by about 4 km to 45±2 km east of Winnipeg (96.51±0.02°W) during 1980–1990 and west again by 2 km to 43±2 km east of Winnipeg (96.54±0.02°W) during 1995–2001. The position of the habitat transition in 1969–1974 was significantly different from all others at α = 0.05, though not from its position in 1948–1957 following sequential Bonferroni correction (Table 2).

### Coincidence

**Figure 5:**
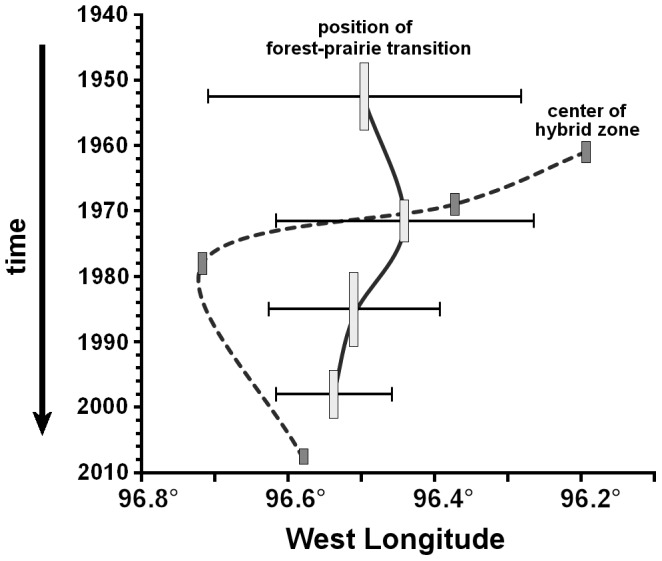
Geographic position (in degrees W longitude) of the centers of the clines in Cranial Crest Separation among toads and the forest-prairie transition in habitat in southeastern Manitoba, 1948–1909. Vertical bar length represents the time covered by each sampling period. Dark bars represent the center of the toads’ hybrid zone, connected by a dashed line. Light bars represent the center of the forest-prairie transition, connected by a solid line. Horizontal bars indicate the estimated width of the forest-prairie transition.

At no time was the position of the cline in toad cranial crest morphology coincident with the position of the forest-prairie transition except, by inference, for a brief moment sometime around 1970 (Fig. 4). Before then, the center of the hybrid zone lay to the east of the forest-prairie transition and, afterwards, it lay to the west. The hybrid zone in 2007–2008 was estimated to lie only 3 km west of the forest-prairie transition in 1995–2001 (*P* = 0.151) but, except for this most recent collection of toads and most recent map, the location of the hybrid zone was not significantly coincident with the cline in forest cover nearest in time (*P*≤0.001; Table 2). In 1960–1962 and 1977–1979, it strayed beyond the estimated width of the forest-prairie transition (Fig. 5).

## Discussion

Over a period of some 50 years, the cline between *A, americanus* and *A. hemiophrys* in southeastern Manitoba not only changed its position significantly and relatively rapidly but also showed distinct evidence of oscillating back and forth. Bidirectional movement like this has not been documented previously in a hybrid zone [1].

Though displaced westward by 28 km between 1960–1962 and 2007–2009, the *A. americanus* x *hemiophrys* hybrid zone actually moved at least 48 km in total west and east over about the same number of years. The extent of this movement is considerably greater than previously thought for this hybrid zone. Cook [18], not suspecting the cline could move, pooled his samples from 1960–1962 with those from 1968–70. In so doing, he placed the cline’s center precisely midway between the positions we determined for those separate data sets. Consequently, Green and Pustowka [7] also appear to have misjudged the magnitude of the cline’s movement. Our concentration on variation in one diagnostic trait, rather than multiple traits simultaneously through use of a discriminant function [7], [18], [19], may also have contributed to the differences between our findings and previous analyses since a discriminant function can mask individual trait variation in merging the variation in multiple traits into a single value [21].

The hybrid zone between these toads is evidently capable of rapid movement. During the first two intervals of study, 1960–1962 to 1968–1970 and 1968–1970 to1977–1979, the cline moved westward by approximately 1.6 km/year and 2.8 km/year, respectively. This may seem astonishing for a toad, though is clearly not impossible considering the dispersal abilities of amphibians [34]. We may, though, have underestimated the cline’s total movement since the third interval of our study, 1977–1979 to 2007–2009, during which the cline appeared to move 10 km east, spanned about 30 yrs. Over that period of time, the hybrid zone may have moved considerably without detection.

Our evidence of oscillation in the position of the *A. americanus* x *A. hemiophrys* hybrid zone offers a new perspective into such a zone’s underlying mechanisms. Although hybrid zones are often found to coincide with habitat clines [35], [36], the lack of coincidence between the centers of the toad hybrid zone and the forest-prairie transition zone, and their respective movement patterns, indicates that the oscillatory movement we have detected is more likely related to such intrinsic factors as relative population size and dispersal, than to changes in habitat [4], [37]. Yet the structure of the hybrid zone is not what would be expected of a moving, density-dependent tension zone [7], [18], [38]. This indicates that an ecotonal hybrid zone may also be affected by the population level process of relative abundance and immigration that are thought to drive movement of tension zones [1], [10], [12]. The forest-prairie habitat transition extends from Manitoba south to the coastal plain of the Gulf of Mexico and is associated with the boundaries between closely related species of many different animals and plants [39], [40], as well as toads. Understanding exactly what may be driving movement in any one of these interactions will contribute towards understanding the dynamics of other hybrid zones in the same region as well as hybrid zones in general.

In conclusion, we have not precisely satisfied the expectations of either of our hypotheses. The hybrid zone clearly has moved but it has not continued to move in the same direction. Furthermore, although it appears constrained on the large scale to the vicinity of the forest-prairie habitat transition, the hybrid zone evidently can, and does, move a considerable distance beyond it, with much more leeway than expected. A variety of drivers may underlie this dynamic [3], [35], [36], [38] but the considerable distance that its center may be located away from the habitat transition indicates that it can be pushed or pulled by demographic pressures to either side. This does not mean, however, that habitat is not important or that the various models proposed for hybrid zones are necessarily exclusive [41]. The toads’ hybrid zone still remains in close proximity to the ecotone. Yet it appears to be not particularly “bounded” by the habitat transition. Rather, it appears to be tethered to it with a fair degree of elasticity.

## Supporting Information

Appendix S1 **Specimens examined. CMNAR = Canadian Museum of Nature, Amphibian and Reptile Collection, RM = Redpath Museum, McGill University, FWS = F.W. Schueler (Canadian Museum of Nature).** Specimens collected as vouchers and used to examine artifact of shrinkage in preservative are indicated with an asterisk (*).
(DOC)
