# Supplementary material for: Oscillation of an Anuran Hybrid Zone: Morphological Evidence Spanning 50 Years
Source: PLoS One. 2012 Dec 26;7(12):e52819. doi: 10.1371/journal.pone.0052819 (PMC3530495; doi:10.1371/journal.pone.0052819)
Supplement: Appendix S1 — Specimens examined. CMNAR = Canadian Museum of Nature, Amphibian and Reptile Collection, RM = Redpath Museum, McGill University, FWS = F.W. Schueler (Canadian Museum of Nature). Specimens collected as vouchers and used to examine artifact of shrinkage in preservative are indicated with an asterisk (*). (DOC) [file pone.0052819.s001.doc]

# Oscillation of an anuran hybrid zone: Morphological evidence spanning 50 years.

# Jean-Sébastien Roy, David O’Connor and David M. Green

**Supporting Information**

# Appendix S1. Specimens examined. CMNAR = Canadian Museum of Nature, Amphibian and Reptile Collection, RM = Redpath Museum, McGill University, FWS = F. W. Schueler (Canadian Museum of Nature). Specimens collected as vouchers and used to examine artifact of shrinkage in preservative are indicated with an asterisk (*).

Reference samples

***Anaxyrus hemiophrys* – Alberta** : CMNAR 10332[1-12], 51.133°N: 114.05°W. **Saskatchewan** : CMNAR 18128[1-14], 54.23°N: 105.92°W; CMNAR 18125[1-15], 54.70°N: 105.77°W; CMNAR 18133[1,3-5], 54.70°N: 105.77°W; CMNAR 4007[1,5,7-9, 11,13,23,25 + 21 lost tags], 50.68°N: 105.57°W; CMNAR 16091[FWS3830-3831, FWS3832], 50.70°N: 103.63°W.

***Anaxyrus americanus* – Ontario** : CMNAR 24449[1-29], 50.80°N: 91.48°W; CMNAR 24443[2-20], 50.53°N: 90.52°W. **Québec** : CMNAR 17097[1,2,4,6,8,9,14,16,18-19,20,24-25,28,31,32,34,36-38,41,43-44, 46,48-50,52-53,57], 45.10°N: 74.385°W; CMNAR 17090[2-8,10-11,18,21-22,25-26, 28,34,36-37,42,47,52-53,55,59,61,63-64,66,67-69], 45.35°N: 73.85°W

Hybrid zone samples.

***Anaxyrus hemiophrys* – Manitoba** : CMNAR 4918[1-8,11,26 + 1 lost tag], 49.62°N: 100.27°W; RM 5038-5041, 49.67°N: 99.55°W; RM 5037, 49.56°N: 99.51°W; CMNAR 4563[1-9,11,13,20,23,27,29,31,35,52,56-57,59,65,66-69 + 9 lost tags], 49.92°N: 99.37°W; CMNAR 12114 49.70°N: 99.08°W; CMNAR 33878[1-5], 49.83°N: 98.97°W; CMNAR 4934[1-7], 49.37°N: 98.84°W; CMNAR 4937[1-3], 49.221°N: 98.80°W; CMNAR 4546[1-3], 49.65°N: 98.47°W; CMNAR 4549[1-3], 49.65°N: 98.44°W; CMNAR 33879[1-6], 50.1843°N: 98.32°W; CMNAR 5388[1,3,4,12,15, 49,50,55,56,58-59,68,74-79,86,87,90,100, 105,117,148,155,170,200 + 9 lost tags], 50.18°N: 98.32°W; CMNAR 5390[3 lost tags], 50.18°N: 98.32°W; CMNAR 4552[1-3], 49.66°N: 98.28°W; CMNAR 3184[5-7,9], 50.15°N: 98.25°W; CMNAR 4525[2,194, 201,204,207-208,215-216,220,224,233,237-238,245,255,259, 269,289,295,299,300-301,305 + 7 lost tags], 50.15°N: 98.25°W; CMNAR 4556[1-3], 49.66°N: 98.23°W; CMNAR 4558[1,3-5 + 4 lost tags], 49.66°N: 98.214°W; CMNAR 4538[4,5 + 3 lost tags], 49.97°N: 98.19°W; CMNAR 9895[1-6], 49.97°N: 98.10°W; CMNAR 4560[4,6,10,2 + 18 lost tags], 49.68°N: 98.06°W**;** CMNAR 4515[1-5], 49.93°N: 97.55°W; CMNAR 4520[2-4,10-12, 14-15,20,21,23-24,27,29-32,35 + 12 lost tags], 49.93°N: 97.55°W.

***Anaxyrus “ americanus x A. hemiophrys”* – Manitoba** : CMNAR 4947[2,3], 49.27°N: 97.35°W; CMNAR 12246[1-12], 49.09°N: 97.28°W; CMNAR 12247[1-7], 49.09°N: 97.28°W; CMNAR 12248[1-7], 49.08°N: 97.27°W; CMNAR 4482[1-8 + 7 lost tags], 50.13°N: 97.18°W; RM 5052-5054*, 49.99°N: 97.16°W; CMNAR 4494[1-4], 49.78°N: 97.13°W; CMNAR 12243[1-4], 49.65°N: 97.13°W; CMNAR 21729[1-6], 49.55°N: 97.03°W; CMNAR 12231[1-18], 49.65°N: 96.98°W; CMNAR 12232[1-3], 49.65°N: 96.98°W; CMNAR 12233[1-9], 49.65°N: 96.98°W; RM 5049-5051*, 49.78°N: 96.84°W; CMNAR 21835[1-12], 50.05°N: 96.80°W; CMNAR 21847[1-2], 50.05°N: 96.80°W; CMNAR 11071[1-15], 50.26°N: 96.73°W; CMNAR 12259[1-5], 49.40°N: 96.72°W; CMNAR 11865[1-4], 49.41°N: 96.69°W; CMNAR 12258[1-12], 49.41°N: 96.69°W; CMNAR 11077[1-13], 50.33°N: 96.65°W; CMNAR 11790[1-8], 49.68°N: 96.60°W; CMNAR 11811[1-12], 49.68°N: 96.60°W; CMNAR 11832[1-5], 49.68°N: 96.60°W; CMNAR 11765, 49.68°N: 96.59°W; CMNAR 11778[1-3], 49.68°N: 96.59°W; CMNAR 11831[1-3], 49.68°N: 96.59°W; CMNAR 6088[1,2], 49.68°N: 96.59°W; CMNAR 6104[1-11], 49.68°N: 96.59°W; CMNAR 12204[1,2], 49.67°N: 96.58°W; CMNAR 12215[1,2], 49.67°N: 96.58°W; CMNAR 12217[1,2,4-6], 49.67°N: 96.58°W; CMNAR 11789[1-3], 49.67°N: 96.57°W; CMNAR 11810[1-9], 49.67°N: 96.57°W; CMNAR 11812[1], 49.67°N: 96.57°W; CMNAR 11815[1,2], 49.67°N: 96.57°W; CMNAR 11840[1-3], 49.67°N: 96.57°W; CMNAR 21817[1-12], 50.05°N: 96.57°W; CMNAR 11788[1-11], 49.68°N: 96.56°W; CMNAR 11039[1-12], 50.46°N: 96.54°W; CMNAR 11254[1-3], 50.07°N: 96.50°W; RM 5046-5048*, 49.89°N: 96.49°W; CMNAR 21816[1-18], 50.05°N: 96.43°W; CMNAR 12219[1,2], 49.67°N: 96.42°W; CMNAR 12221[1-5], 49.67°N: 96.42°W; CMNAR 12222[1-15], 49.67°N: 96.42°W; CMNAR 21889[1-3], 49.66°N: 96.41°W; CMNAR 11018[2,3,5,9,13,17-18,21,23-24,28,32,33, 35-38,40,45,48,49,51,53-55,59,62-63,70,71], 50.05°N: 96.40°W; CMNAR 11026[1-15], 49.67°N: 96.38°W; CMNAR 11029[1-3,5-7,9-15,17-34], 49.88°N: 96.37°W; CMNAR 21836[1-10], 49.66°N: 96.311°W; CMNAR 10992[1-3], 49.67°N: 96.31°W; CMNAR 10994 49.65°N: 96.28°W; CMNAR 10998[1,2], 49.65°N: 96.28°W; CMNAR 11013[1-27], 49.65°N: 96.28°W; CMNAR 11024[1], 49.65°N: 96.28°W; CMNAR 11025[1-19], 49.65°N: 96.28°W; CMNAR 11064[1-3], 49.21°N: 96.28°W; CMNAR 11028[1,2,4,9,10-15, 19,21,24-25,28,29,31,33-36,38,40,42,46-47,49,50,52-53], 49.88°N: 96.28°W; CMNAR 21833[1-15], 49.66°N: 96.25°W; RM 5045*, 49.66°N: 96.24°W; CMNAR 11528 50.06°N: 96.20°W; CMNAR 11019[2,5,6,8,11, 21-23,25-29,32-37,39-41,43-46,48-50, 52], 50.06°N: 96.20°W; CMNAR 4500[48,54,104,105], 49.23°N: 96.20°W; CMNAR 21731[1-7], 49.66°N: 96.15°W; CMNAR 11802[1-25], 49.66°N: 96.14°W; CMNAR 11854[1-4], 49.67°N: 96.08°W; CMNAR 5350[1-18,20,22-24,26,28,29,32-35 + 3 lost tags], 49.65°N: 96.01°W

***Anaxyrus americanus* – Manitoba** : CMNAR 10996[3,8,12,15-17,19,20,22,27, 30,31,34, 37-40, 42,44-46,49,52,55,59,60,67,71-73], 49.66°N: 95.99°W; CMNAR 11775[1-15], 49.66°N: 95.99°W; CMNAR 11787[1-20], 49.66°N: 95.99°W; CMNAR 12200[1-19], 49.66°N: 95.99°W; CMNAR 21840[1-7], 49.67°N: 95.98°W; CMNAR 11042[1,3,4,6-17,19,21,22,24-36,38], 49.66°N: 95.97°W; CMNAR 11807[1-21], 49.66°N: 95.97°W; CMNAR 11012[1-13,16,18-24,26,31,34-36,40-63,51,53], 49.91°N: 95.95°W; CMNAR 11067[1-9], 49.07°N: 95.93°W; RM 5042*, 49.65°N: 95.91°W; CMNAR 21843[1-11], 49.67°N: 95.85°W; RM 5043*, 5044, 49.64°N: 95.64°W; CMNAR 11011[1,2,4-7,9-11,13,15,19,23,26,30,32, 37,39,45-51,61-63,66,67], 49.85°N: 95.46°W; CMNAR 21834[1-6], 49.70°N: 95.25°W; CMNAR 6054[11,13, 32,35,39, 43,44-45,46-57,63,66-67?,69,77,79,82,88,93-94,98-100,106-108,111,113 + 1 lost tag], 49.70°N: 95.23°W; CMNAR 11002[1-16], 49.78°N: 95.22°W; CMNAR 21829[1-12], 49.72°N: 94.93°W; CMNAR 21874[1-6], 49.72°N: 94.93°W
